# Screening of Oomycete Fungi for Their Potential Role in Reducing the Biting Midge (Diptera: Ceratopogonidae) Larval Populations in Hervey Bay, Queensland, Australia

**DOI:** 10.3390/ijerph8051560

**Published:** 2011-05-13

**Authors:** Kirsty Stephen, D. Ipek Kurtböke

**Affiliations:** Faculty of Science, Health and Education, University of the Sunshine Coast, Maroochydore DC, QLD, 4558, Australia; E-Mail: kirsty.stephen@gmail.com

**Keywords:** biting midge, *Culicoides subimmaculatus*, oomycete fungi, *Halophytophthora*, biological control

## Abstract

Biting midges are globally distributed pests causing significant economic losses and transmitting arbovirus diseases to both animals and humans. Current biological and chemical control strategies for biting midge target destruction of adult forms, but strategies directed at immature stages of the insect have yet to be explored in Australia. In the present study, coastal waters of Hervey Bay region in Queensland, Australia were screened to detect the habitats of biting midge at immature stages. These results were then correlated to local environmental conditions and naturally occurring entomopathogenic fungal flora, in particular the Oomycete fungi, to determine their reducing effect on insect immature stages in the search for biological control agents in the region. The dominant species of biting midge found within this study was *Culicoides subimmaculatus* occuring between mean high water neaps and mean high water spring tide levels. Within this intertidal zone, the presence of *C. subimmaculatus* larvae was found to be influenced by both sediment size and distance from shore. *Halophytophthora* isolates colonized both dead and alive pupae. However, the association was found to be surface colonization rather than invasion causing the death of the host. Lack of aggressive oomycete fungal antagonists towards midge larvae might correlate with increased incidences of biting midge infestations in the region.

## 1. Introduction

Biting midges are common pests, found in many different countries and regions such as the USA, the Caribbean, Africa, Mexico, Canada, Scotland and Australia [1–3]. Australia has at least 70 species from the genus *Culicoides* [4], including *C. ornatus*, found extensively within Northern Territory [5]; *C. molestus* [6,7] and *C. subimmaculatus* affecting South East Queensland [8]. *Culicoides* species are globally responsible for transmitting viral diseases of livestock and wild ruminants, such as African Horse Sickness Virus, Bluetongue Virus (*BT*V), Epizootic Hemorrhagic Disease Virus, Palyam Viruses, Equine Encephalosis Virus, Bovine Ephemeral Fever Virus (BEFV), and Akabane Virus [9]. *Culicoides* species are also suspected vectors of vesicular stomatitis virus, causing significant economic losses in cattle, horses and swine [10] and are reported to transmit diseases to native wildlife including *BTV* to wild ruminants [11,12], hemorrhagic disease in white-tailed deer [13], and blood parasites within birds [14].

Of the two common South East Queensland species, *C. subimmaculatus*, is associated with the surface-tunneling crab *Mictyris livingstonei* McNeill, and found between mean high water neap (MHWN) and mean high water springs (MHWS) [6] in areas of sand or sand-mud with minimal wave action [15]. *C. molestus* is found breeding along canal beaches in fairly clean flocculated sand between mean tide levels (MTL) and MHWS [7,16].

Residents in midge prone areas have limited options to control invading species [15], with persistent attacks by swarms significantly impacting on work and recreational activities [17]. Due to adult biting-midge dispersal from their original source, treatments targeting adult populations are usually ineffective [18]. Immature stages of *Culicoides* species are more susceptible to treatment [18], however, the costal species are located within the sensitive inter-tidal zones along beaches, estuaries and waterways out of easy reach [19].

Possible treatment options for the control of biting midge can include larviciding, adult insecticidal fogging, barrier treatments or habitat modification. Adult insecticidal fogging is the application of aerosol particles against flying insects [20]. However, this method is usually ineffective and short-term [15] due to rapid re-infestation, limited accessibility to treat large areas, and the significant costs involved [20]. Low specificity, emerging resistance, pesticide side-effects, toxic derivatives and long term environmental pollution [21] from pesticide use have highlighted the need for non-polluting and safe pest controls such as the use of biological control agents. Advantages of biological control agents over chemicals are numerous, including safety of non-target species [22], increased activity of natural enemies and increased biodiversity [23].

The majority of insect orders are susceptible to pathogenic fungi [24] with populations commonly affected by extensive epizootic disease events [25]. Entomopathogenic fungi are important regulators within insect populations, synchronization with the host’s lifecycle is commonly seen [26] and diverse ranges of strategies from obligate parasitism to opportunistic pathogens have been observed [23].

Many of the Oomycetes including *Pythium*, *Phytophthora*, *Saprolegina*, *Aphanomyces*, and *Lagenidium* are parasites of plants or animals using zoospores to infect their hosts [27]. Dominant marine Oomycetes are species of *Halophytophthora* [28], which are probable decomposers of fallen leaves [28]. Investigations indicate that species of *Halophytophthora* are widespread in mangrove communities in southern coastal Queensland [29]. The Oomycete *Lagenidium giganteum* is a facultative fungal pathogen of mosquito larvae [30] and has been registered for use as a mosquito biological control agent with the United States Environmental Protection Authorities [27,31], and successfully controls mosquitoes from the genera *Anopheles*, *Aedes*, *Culex*, *Culiseta* and *Psorophora* [31].

In Australia, past research has identified two fungal species entomopathogenic to biting midge larvae. Naturally occurring fungal species in Northern New South Wales, *L. giganteum* produced mortality rates of up to 33% against *C. molestus* larvae [7]. A study by Unkels *et al.* [1] has also shown *Culicinomyces clavosporous* to be highly pathogenic to the larvae of *C. nubeculous* [1]. The study reported here is a further investigation into the presence of naturally occurring entomopathogenic oomycete fungi against biting midge larvae within Hervey Bay, Queensland, in order to assess their potential as biological control agents and a safe alternative to chemical control in the region.

## 2. Experimental Section

### 2.1. Sampling Sites

Sampling was conducted at four locations within Hervey Bay, including River Heads, Urangan, Eli Creek and Beelbi Creek (Table 1). Sampling locations were chosen due to the known presence of adult biting midge within the area and to ensure that samples were derived from both open beach, estuarine intertidal and creek environments. Table 2 lists *Halophytophthora* isolates found in Harvey Bay.

At each transect site, environmental parameters included sediment characteristics, mangrove density and the distance at which larvae occurred within the tidal plain to identify any significant associations with biting midge larval habitats and these parameters within Hervey Bay [32]. Other environmental variables such as water salinity, temperature and pH were also measured to describe their effect on the larval and fungal habitats (Table 3).

The association between larvae abundance and mangrove density was also tested in a single transect taken longitudinally along Eli Creek mouth, where changes in mangrove density could be measured in relation to biting midge larvae being present. The sorting of sediments fell into five categories from “very well sorted” to “poorly sorted” and their relationship to larval presence was analyzed (sediment sorting is the measure of the range of grain sizes and spread of these sizes around the mean particle size within each sediment sample [33]).

### 2.2. Larval Sampling and Extraction

Samples were collected by transecting the intertidal zones from above mean high water springs (MHWS) to beyond mean high water neap (MHWN) at low tide. Larvae were sampled by digging a channel 1 m long, 4 cm deep, and 4 cm wide with a hand shovel at measured points along each transect. Points sampled for larvae were dictated by the overall distance covered by each individual transect, and ranged from one to twenty meters apart. Samples were stored within large zip-lock plastic bags, and kept at room temperature in the shade, for up to 48 hours before processing.

Larvae were then extracted using two different separation methods. Sugar-flotation method was used for samples composed of sands or sand-silt substrates, whilst a salt flotation method [34] was employed for samples with higher silt/clay proportions. When using sugar flotation, the sample was flooded and mixed with a 2:8 parts molasses water mix, and then allowed to settle for 30 minutes in 1 L plastic measuring cylinders. After settling, the top liquid layer was first sieved through a large generic sieve, then a fine (63 micron) sieve. The fine sieve was then rinsed out into a clear plastic holding container with tap water to extract the larvae. Salt flotation followed the same method as sugar flotation however, a ∼40% w/v Epsom Salt solution was used with all samples settled and sieved twice before discarding. Extracted larvae were refrigerated in sealed containers of tap water, with counting and identification completed within 24 hours after extraction.

### 2.3. Larval Identification

Methods for enumeration and identification of larvae were obtained from the Tweed Heads Shire Council. Holding containers of larvae were placed over a black ceramic tile and the larvae excited into swimming by using a strong light source and a plastic bulb pipette to disturb any sediment present. Excited larvae were then easily identifiable by their distinct swimming pattern, and white coloration against the black background.

After counting, larvae were stored within a refrigerator at 4 °C for up to 12 hours to initiate a state of immobilization resulting from the decreased temperature. Identification was then conducted on immobilized larvae by observing head struts and pigmentation patterns along the thoracic region of the insect body. Any larvae displaying discoloration were isolated for comparison with healthy larvae to determine if a fungal infection was present. These discolored larvae were later used to attempt isolation of fungal isolates.

### 2.4. Fungal Isolations and Identifications

Samples for fungal isolations were taken from transects within each of the sampled four locations within Hervey Bay (Table 1). Samples taken along each transect included water, composite samples of leaf matter and sediment. Leaf and sediment samples were collected and stored in zip-lock plastic bags whilst water samples were stored in sterile 100 mL screw top containers. All samples were kept on ice for transport and refrigerated at <4 °C while in storage.

For isolations, both direct and conventional serial dilution techniques were used. For direct isolations of fungi three individual leaves of varying decomposition were selected and surface sterilized subsequently [35]. After surface sterilization, each leaf was dissected into small squares using a sterile scalpel and aseptically placing onto 3P agar, selective for *Phytophthora* and other oomycete species [36]. A serial dilution, adapted from Sylvia *et al.* [37], was performed on both the water and sediment samples. Before diluting, sediment samples were shaken for 20 minutes with a Griffin Shaker and water samples vortexed to create a homogenous sample, with aliquots from selected dilutions plated.

Fungal isolation was also carried out from larvae displaying symptoms of possible fungal infection. Larvae were surface sterilized by soaking in a 5% bleach solution for 60 seconds and rinsed in sterile water before plating onto 3P Agar. All inoculated plates were incubated at 22 °C, in the dark, and checked periodically over a 14-day period. After initial isolation on to 3P media, the resulting fungal colonies with oomycete mycelial growth characteristics were subcultured onto Potato Dextrose Agar (PDA) or V8 agar [34] for purification, and resulting pure cultures were identified and stored under oil for preservation. For identifications, fungal isolates with white mycelia presenting distinct Oomycete growth patterns [38] were selected. Samples were prepared for microscopic examination by scraping off a small amount of mycelia and placing it in a drop of lactoglycerol solution (lactic acid, 25 mL; glycerol, 50 mL; distilled water, 25 mL) on a microscope slide [39]. The samples were then observed for presence of coenocytic hyphae, a morphological characteristic of *Phytophthora* and *Halophytophthora* [31]. Once coenocytic isolates were identified, isolates were flooded with seawater to induce sporangia and zoospore production for identification of *Halophytophthora*.

### 2.5. Insecticidal Bioassay

Two different bioinsecticidal assays were used to assess the antagonistic potential of the oomycetes isolated from Hervey Bay biting midge habitats against the biting midge *Culicoides subimmaculatus*. Other oomycete fungi obtained from DPI, Orange, NSW, Australia were also included in the study to determine whether they would possess any insecticidal properties should local isolates fail to prove to be antagonistic (Table 4).

A larval colonisation bioassay adapted from Sweeney [40] and an insecticidal metabolite bioassay adapted from Sur *et al*. [41] were used to test the presence of antagonistic activity by the fungal cultures.

A *Dialysis Membrane Overlay Technique* [42] was also used to detect any diffusible antifungal compounds that might be produced by the isolates.

### 2.6. Statistical Analysis

A chi-square test for independence, performed on SPSS version 14.0 for windows, was used to determine any association between the presence/absence of oomycete fungi and biting midge larvae.

## 3. Results

### 3.1. Detection of Larvae

**Figure 1.**
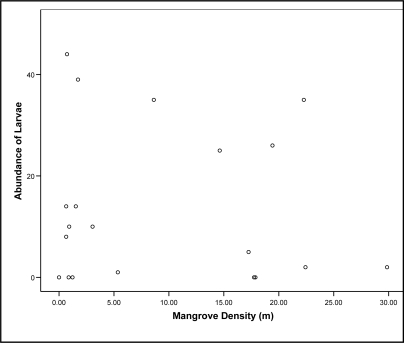
The abundance of larvae relative to mangrove density (m).

Figure 1 displays a scatter plot of larval abundance compared to the resulting mangrove densities measured. Larvae were found in moderate to high numbers over a large range of mangrove densities. A Kruskal-Wallis test found no significant association, between the distance within the ripple/mud plain and the abundance of larvae, present (χ^2^ = 2.808, p ≥ 0.05).

**Figure 2.**
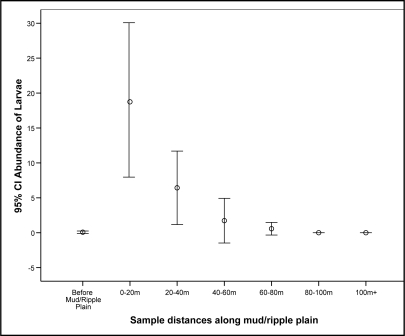
Sample distances within intertidal zones along the mud or ripple plain where biting midge larvae populations were located.

Furthermore, the larvae were found in large numbers within the 0–20 m zones over the sandy and ripple plains. A *Kruskal-Wallis* test showed that there was a significant association between the distance within the intertidal zones sampled and the abundance of larvae present (χ^2^ = 53.894, p ≤ 0.001). A chi square test for independence showed there was a significant association between larvae presence/absence with distance zones over the sandy ripple plain (χ^2^ = 71.752, p ≤ 0.001) (Figure 2).

**Figure 3.**
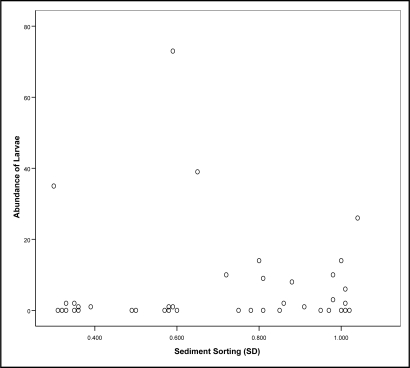
The sediment standard deviation, expressed as *Phi*, of samples from Eli Creek and Urangan in comparison to the abundance of larvae found within each sample.

Figure 3 shows a scatter plot of sediment sorting compared to the number of larvae found. A *Kruskal-Wallis* test showed no significant difference between the abundance of larvae and the sorting of sediments (χ^2^ = 0.482, p ≥ 0.05).

### 3.2. Fungal Isolations and Identifications

**Figure 4.**
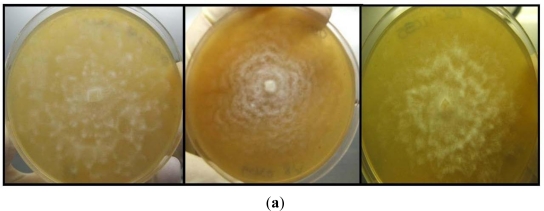

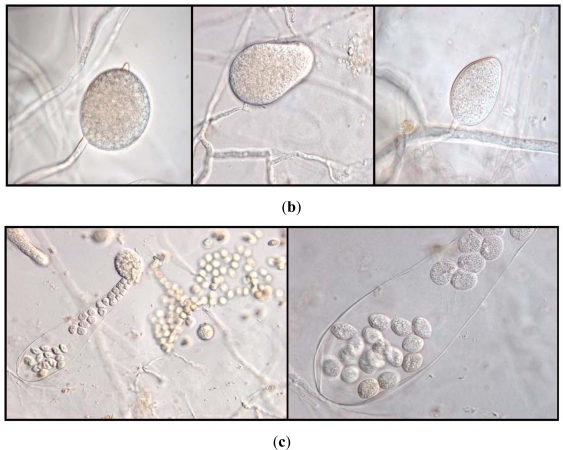
(**a**) Growth patterns of *Halophytophthora* isolates; (**b**) Sporangia produced by some of the *Halophytophthora* isolates; (**c**) Zoospores of a *Halophytophthora* isolate.

Twenty-six marine *Halophytophthora* isolates were isolated from Hervey Bay; all resulting from leaf samples of varying decomposition, collected throughout individual transects (Table 2, Figures 4a, b, and c). *Halophytophthora* isolates were detected mostly in environments with median salinity of 33.16 ppt, temperature of 22.38 °C, and a pH of 7.78 (Table 3).

*Halophytophthora* isolates were obtained from 50% of the sites where larvae were detected and from 31.3% of sites where larvae were not detected. There was no significant association between the existence of *Halophytophthora* and the existence of biting midge larvae (Table 5). A chi square test for independence showed no significant association between *Halophytophthora* and larval existence (χ^2^ = 0.482, p = ≥ 0.05).

### 3.3. Bioassays

In both insecticidal bioassays, larvae matured through to pupae with a small number maturing into adults, indicating absence of true antagonistic activities for all isolates listed in Table 2. All of the isolates tested were associated with both dead and alive pupae; however, the association appeared to be through surface colonization and/or attachment, but not invasion causing death of the host. Isolates tested resulting in observed mycelial growth and attachment to pupae are listed in Table 4 including the isolate USC-021-C4 which resulted in mycelial growth around thoracic segments of two pupae cadavers. Furthermore, no insecticidal activity was present when a diffused fungal metabolite test was performed, with all larvae remaining alive and active throughout the 14-day insecticidal bioassay test period. A large number of larvae matured into pupae, with some then maturing into adult midge by day 14. Both test and control larvae survived remaining alive and active throughout the test period.

## 4. Discussion and Conclusions

Species of oomycete fungi in particular *Halophytophthora* were targeted for isolations in this study as they were reported to occur within the marine intertidal environment [28] and belong to the Family *Pythiaceae* (Class Oomycetes), from which, *Lagenidium giganteum*, biting midge larvae infecting fungus was previously described as a biological control agent [7].

*Halophytophthora* are the first colonizers of mangrove leaves [28] essential within the mangrove ecosystem [43] and are significant decomposers of leaf litter [28,43]. If an entomopathogenic species of *Halophytophthora* associated with the presence of leaf litter and/or specific mangrove species were identified, such a finding could have significance in the search for biological control agents in these environments.

However, no naturally occurring antagonists were detected in this study at the locations of populations of biting midge larvae which may also explain the high numbers of biting midge within the region [44,45]. There was also no association between the *Halophytophthora* species isolated and the larval occurrence at the sites sampled. Nakagiri [46] suggests that species of *Halophytophthora* occur when and where environmental conditions suit them. Mangrove ecosystems vary greatly in water salinity [43], and pH concentration [43,47]. They also experience temperature changes, which can vary between 10–20 °C within the 24-hour tidal cycle [48]. The detection of fungi at temperatures around 20 °C but not above might suggest unsuitable environmental conditions supporting fungal growth in the zone such as higher temperatures at this sub-tropical region.

Further investigation into the relationship between sediments and larval populations over a broader range of habitats, including the influence of mangroves on sediment accumulation within the intertidal environments, might provide further information and contribute to the identification of processes resulting in the accumulation of sediments preferred by biting midge larval populations, or lead to the identification of a method to deter larval populations through sediment manipulation.

In order to be able to design effective biocontrol measures, a thorough understanding is required of the natural habitat and ecology of the antagonistic species so that their suitability as a biocontrol agent can be determined. Naturally occurring fungal species, other than Oomycetes, occurring within intertidal zones could also be investigated to determine whether previously undetected bioinsecticidal fungi may be present within these locations. Alternatively, previously identified antagonistic species such as the Northern New South Wales isolate *Lagenidium giganteum* (producing mortality rates of up to 33% against *C. molestus* larvae [7]) or *Culicinomyces clavosporous* (shown to be highly pathogenic to the larvae of *C. nubeculous* [1]) might also be considered for bio-reinforcement/bioaugmentation strategies for elimination of the biting midge larvae in the infested areas of the region.

The identification of larval habitats within this study is not complete for the Hervey Bay region however; findings of the study may assist future locating, surveying and/or controlling of larval habitats and their natural predators within this area in order to design effective biological control measures to replace currently used environmentally unfriendly chemicals.

## Figures and Tables

**Table 1.** Summary of transects sampled within the four study locations of Hervey Bay.

**Location**	**Transect**	**Type of location**	**Substrate type**	**Vegetation**	**Mictyris Crab**	**Larvae**

**River Heads**	007	Open Beach (West)	Mud-flat	Minimal mangrove fringe	Present in sandy area	0
	019	Open Beach (East)	Sand/mud zone, followed by mud-flat to water’s edge	Dense mangrove forest	Present throughout inner zone of mangrove forest	44
	020	Open Beach (West)	Short sandy-flat rapidly changing into a mud-flat	Moderately dense mangrove fringe	Present in sandy area	196
	021	Open Beach (West)	Short sandy-flat rapidly changing into a mud-flat	Moderately dense mangrove fringe	Present in sandy area	75

**Urangan**	001	Open Beach	Short coarse sand-flat moving into rocky plain	None	Absent	0
	002	Open Beach	Short coarse sand-flat moving into rocky plain	None	Absent	0
	003	Open Beach	Sandy/Mud-flat	Moderately dense mangrove fringe	Minimal within mangrove fringe	0
	018	Tidal Creek	Sandy tidal creek beach fore-dune	Mangroves along edge of creek	Dense throughout transect	19
**Eli Creek**	004	Open Beach	Rocky shore followed by extensive sandy flat	None	Absent	1
	006	Estuarine	Deep mud	Dense mangroves surrounding site	Absent	0
	008	Open Beach	Coarse sandy beach with large ripple plain	None	Absent	0
	009	Open Beach	Sand-flat with mud increasing in presence	Scattered mangroves throughout mud-flats	Absent	9
	010	Estuarine	Mud-flats with moderate sand present	Moderately dense mangrove presence throughout mud-flats	Extensive, close to start of mud-flat, scattered throughout	110
	011	Estuarine	Mud-flats with moderate sand present	Scattered mangroves throughout mud-flats	Extensive, close to start of mud-flat, scattered throughout	102
	012	Estuarine	Mud-flat	Dense mangrove forest throughout mud-flats	Scattered	25
	013	Estuarine	Steep short mud-bank	Scattered mangroves present	Scattered throughout	15
	014	Estuarine	Steep short mud-bank	Moderately dense mangroves present	Scattered throughout	55
	015	Estuarine	Island within Eli Creek, mud-flats	Moderately dense mangroves present	Absent	133
	016	Estuarine	Large meander bend flanked by Eli Creek, mud-banks	Four distinct zones of mangrove succession, dense mangrove presence	Some present	304
	017	Estuarine	Transition of sand to mud-flats	Mangroves changing in density throughout transect	Extensive, close to start of mud-flat, scattered throughout	1202

**Beelbi**	005	Estuarine	Sandy mud	Mangrove fringe	Absent	0

**Table 2.** *Halophytophthora* isolates from Hervey Bay.

**Location**	**Transect**	**Isolate numbers**

**River Heads**	019	USC-019-A2; USC-019-A3; USC-019-A4; USC-019-C1; USC-019-C2; USC-019-C3
	020	USC-020-A3; USC-020-C1; USC-020-C2
	021	USC-021-A1; USC-021-A3; USC-021-B2; USC-021-C1; USC-021-C2; USC-021-C4

**Urangan**	001	USC-001-1; USC-001-2

**Eli Creek**	004	USC-004-1
	013	USC-013-A1; USC-013-A2
	014	USC-014-1
	016	USC-016-C1; USC-016-C2; USC-016-B1; USC-016-B2

**Beelbi Creek**	005	USC-005-1

**Table 3.** Details of environmental variables found in sampled locations where *Halophytophthora* were isolated.

**Variables**	**Fungi isolated**		**Fungi not isolated**	
	**Median**	**Range**	**Median**	**Range**

Salinity (ppt)	36.43	29.34–39.01	29.88	16.93–39.01
Temperature (°C)	20.45	19.2–23.4	24.10	19.1–26.8
pH	7.91	7.72–8.11	7.6	7.47–8.11

**Table 4.** Results of larval colonization bioassay.

**Isolate code**	**Effect on the larvae**

Control (no fungal inoculum present in the larval growth environment)	Larvae alive and active
Reference strain-73864(Pythium prolatum)*	Dead pupae, surrounded by mycelia, no invasion or outgrowth visible. Larvae alive
Reference strain-50182(Halophytophthora batemanensis)*	Dead pupae, surrounded by mycelia, no invasion or outgrowth visible. Larvae alive
Reference strain-76023 (Phytophthora gonapodyides)*	Dead pupae with visible mycelia growth (day 3), microscopic examination shows pupae consumed by fungi with significant fungal outgrowth from exoskeleton around the thoracic region. Larvae alive
*Halophytophthora* isolate-USC-005-1	Pupae alive with mycelia growth attached. Larvae alive.
*Halophytophthora* isolate-USC-019-C3	Pupae alive with mycelia growth attached. Larvae alive.
*Halophytophthora* isolate-USC-021-C4	Two dead pupae, mycelia growth around thoracic segments of both pupae. Larvae alive.

* reference oomycete fungi obtained from DPI, Orange, Australia.

**Table 5.** The abundance of larvae found in relation to *Halophytophthora* existence *.

			***Halophytophthora***		**Total**
			**Absent**	**Present**	

Larval sites showing Presence/Absence of larvae	Absent	Count	11	5	16
		% within Presence or Absence of Larvae	68.8%	31.3%	100.0%

	Present	Count	2	2	4
		% within Presence or Absence of Larvae	50.0%	50.0%	100.0%

Total		Count	13	7	20
		% within Presence or Absence of Larvae	65.0%	35.0%	100.0%

* (p = ≥ 0.05).
